# Predicting competitive alpine skiing performance by multivariable statistics—the need for individual profiling

**DOI:** 10.3389/fspor.2024.1505482

**Published:** 2025-01-15

**Authors:** Robert Nilsson, Apostolos Theos, Ann-Sofie Lindberg, Christer Malm

**Affiliations:** ^1^ Section of Sports Medicine, Department of Community Medicine and Rehabilitation, Umeå School of Sport Sciences, Umeå University, Umeå, Sweden; ^2^ Department of Health, Education and Technology, Division of Health, Medicine and Rehabilitation, Luleå University of Technology, Luleå, Sweden

**Keywords:** slalom, peak performance, training, exercise testing, sport performance

## Abstract

**Introduction:**

Predicting competitive alpine skiing performance using conventional statistical methods has proven challenging. Many studies assessing the relationship between physiological performance and skiing outcomes have employed statistical methods of questionable validity. Furthermore, the reliance on Fédération Internationale de Ski (FIS) points as a performance outcome variable presents additional limitations due to its potential unreliability in reflecting short-term, sport-specific performance. These factors complicate the selection of appropriate tests and the accurate prediction of competitive outcomes.

**Method:**

This study aimed to evaluate the predictive power of a generalized physiological test battery for alpine skiing performance, as measured by FIS points, utilizing multivariable data analysis (MVDA). Physiological test results from a total of twelve (*n* = 12) world-class female skiers were included in the analysis.

**Results:**

The result on goodness of regression (R^2^) and goodness of prediction (Q^2^) in this study indicate that valid Orthogonal Projection to Latent Structures (OPLS) models for both Slalom and Giant Slalom can be generated (R^2^ = 0.39 to 0.40, Q^2^ = 0.21 to 0.15), but also that competition performance still cannot be predicted at a group level (low Q2). In contrast, higher predictive power of competitive performance was achieved on an individual level using the same data (R^2^ = 0.88 to 0.99 and Q^2^ = 0.64 to 0.96).

**Discussion:**

The findings of this investigation indicate that the selected tests employed in this study exhibit limited generalizability for the assessment of elite alpine skiers, as the predictive value of specific physiological parameters on competitive performance appears to be highly athlete-dependent.

## 1 Introduction

Physiological testing is widely used in sports to simulate and estimate athletic performance in controlled conditions (1). These tests provide valuable insights into an athlete's physiological status, enabling the assessment of training interventions over time (1, 2) and helping optimize performance in competitive settings (3). For these tests to be practical and meaningful, their protocols must demonstrate both reliability and validity (4, 5). Reliability refers to the consistency or stability of the test outcomes, while validity indicates how accurately a test measures or predicts what it is designed to assess (1). In this context, competitive physical performance serves as the dependent (predicted) variable, while physiological test results function as the independent (predicting) variables.

Predicting alpine skiing performance using common physiological tests has proven challenging (6, 7). The complexity of the sport itself (8–10), coupled with difficulties in establishing reliable response variables (e.g., competitive performance), complicates the identification of valid and reliable tests for predicting success in competition. While several studies (11–14) have identified relationships between anthropometric and physiological test variables and ranked skiing performance, such as Fédération Internationale de Ski (FIS) rankings, many of these studies employed questionable statistical methods (e.g., using parametric instead of non-parametric statistics), undermining the validity of their findings (7).

Additionally, the FIS ranking system itself can be problematic as a performance indicator due to the algorithm used for calculating ranks, which may encourage tactical and opportunistic behavior (15). Other studies have employed alternative performance outcomes, such as time trials (which lack competitive elements) or the number of top-three finishes (dependents on participant strength in each race) (16–18). Each of these response variables has its own limitations and may not accurately reflect long-term competitive performance.

Without a reliable response variable, the selection of physiological tests, while valid in general, may not accurately predict competitive performance (1). This uncertainty can lead to misguided evaluations of training interventions and flawed decisions when selecting athletes for teams or competitions.

Reliable and valid physical tests are essential for optimizing athletic performance.

In various sports, efforts to quantify performance profiles reveal that elite athletes exhibit distinct key performance variables compared to their less skilled counterparts (19, 20). However, significant differences in performance variables are also observed among elite athletes, even those with similar rankings (21). These individual differences among elite athletes significantly affect their physiological and psychological capabilities, training requirements, and performance outcomes (22). Factors such as strength, endurance, mobility, coordination, confidence, and mental stamina can all influence an athlete's overall competitive success. Therefore, training plans and test protocols should be tailored to each athlete's unique needs and goals, depending on the sport and personal ambitions (22, 23).

Individualized training is generally considered essential for maximizing performance, preventing injuries, and maintaining long-term motivation and commitment (24).

However, when implementing physiological tests, clubs, sports organizations, and federations often use generalized protocols to assess athletes’ physiological status. For elite athletes, this approach is likely suboptimal and rarely provides a clear indication of which physiological qualities are critical for sport-specific performance at an individual level.

In the worst-case scenario, these tests could recommend a training regime that is counterproductive to the athlete's competitive abilities.

A significant knowledge gap exists in the identification of valid physical tests that can accurately predict alpine skiing performance and assess the efficacy of training interventions.

The necessity for reliable and valid test batteries in elite sports, along with the development of individualized prediction profiles based on generated results, is crucial.

Therefore, the primary objective of this study is to investigate the predictive power of anthropometric and physiological tests for sport-specific performance in world-class female alpine skiers over time.

A secondary objective is to address the challenges of predicting alpine skiing performance at a group level and to explore the potential for individualized profiling models to enhance the accuracy of performance predictions.

We hypothesis that multivariable statistical method will generate statistically significant, and valid models for prediction of competitive alpine skiing performance.

## 2 Material and methods

### 2.1 Participants

A cohort of twelve (*n* = 12) elite female alpine skiers (age 18–31 years), volunteered to participate in this investigation (Table 1). All participants were active members of the National Swedish Alpine Skiing Team at the time of testing, with a majority holding a top-ranking world status in the disciplines of slalom (SL) and giant slalom (GS). During the pre-competitive phase, each athlete adhered to a structured and individualized training protocol, comprising up to ten weekly sessions that incorporated strength, endurance, mobility, and other performance-enhancing exercises. In the competitive season, athletes engaged in regular racing activities. Prior to conducting the tests, participants were thoroughly informed of the potential risks and discomfort associated with the experimental procedures and provided written informed consent for their participation.

**Table 1:** Anthropometric and physiological data of elite female alpine skiers.

Variable	(*n* = 12)
Body stature (cm)	172.3 ± 5.2
Body weight (kg)	67.9 ± 3.7
Total bone mass (g)	2,985 ± 183
Total fat mass (g)	13,692 ± 2,296
Total lean mass (g)	51,150 ± 2,193
Total tissue mass (g)	64,842 ± 3,206
Total body fat (%)	21.1 ± 2.8
Handgrip strength right (kg)	56.5 ± 6.4
Counter movement jump (cm)	35.1 ± 5.1
V̇O_₂peak_ ergometer cyckling (ml·kg^−1^·min^−1^)	50.3 ± 3
Rowing ergometer 500 m (s)	98.2 ± 3.2
Rowing ergometer 500 m peak power (W)	462 ± 45
Rowing ergometer 500 m mean power (W)	364 ± 32
Estimated V̇O_₂peak_ rowing ergometer 500 m (ml·kg^−1^·min^−1^)	52 ± 3
Lateral box jump 90 s (n)	79 ± 8
Illinois agility test (s)	16 ± 0.5
Y balance test™ anterior H (cm)	62.9 ± 4.8
Y balance test™ anterior L (cm)	62.3 ± 5.0
Y balance test™ posteriomedial H (cm)	104.9 ± 4.5
Y balance test™ posteriomedial L (cm)	105.5 ± 4.9
Y balance test™ posteriolateral H (cm)	104.3 ± 4.4
Y balance test™ posteriolateral L (cm)	103.6 ± 4.9
Force-velocity bike test peak power (W)	878 ± 102
Force-velocity bike test peak power (W·kg^−1^)	12.9 ± 1.5

All variables initially included in statistical analyses.

### 2.2 Ethical statement

Ethical permission 2016-260-31M was granted by the local ethical committee for northern Sweden at Umea University, and the study was conducted in accordance with the ethical principles outlined in the Declaration of Helsinki for Medical Research Involving Human Subjects 2008.

### 2.3 Methodology

The testing and multivariable statistical procedures used in this study are described in previous work by our group (6, 7, 25) and others (26). Methods that deviate from these are described in detail in this section. Data was compiled from body composition measurements and physiological performance tests conducted at the Section of Sports Medicine, Umeå University, Sweden. Each test session was scheduled near the preceding or upcoming race season, corresponding to the periods from April to May and August to October.

The selection of performance tests in the present study was based on our previous research (6, 7, 25), common practice, and the requirement to cover a broad spectrum of physiological qualities. Athletes performed the same test battery multiple times per year over several years.

Repeated measurements are included in statistical analyses.

### 2.4 Anthropometric measurements

Body composition and bone measurements were assessed using a phantom-calibrated Lunar iDXA (GE Medical Systems Lunar, WI, USA; Encore Version 14.10.022). To reduce the risk of redundancy, variables with the strongest weight for predictive models/profiles, were selected by principal component analysis (PCA). After a visual inspection, total fat mass (g), lean body mass (g), tissue mass (g), bone mass, and body fat (%) were selected for inclusion in the statistical analysis.

### 2.5 Physiological testing

During the tests, athletes wore lightweight clothing such as tank tops, T-shirts, shorts, tights, and suitable footwear. Standardized nutrition or pre-test training protocols are not possible in elite athletes, as they follow rigid, individual, schedules. All tests were administered by experienced and trained testing personnel (RN, CM, AT).

A battery of tests was employed to evaluate the participants’ physiological performance. These tests included maximal handgrip strength, countermovement jump (CMJ), 500 m ergometer rowing for approximation of peak oxygen consumption (V̇O_2peak_) and measure of mean power, Illinois Agility Test (IAT) for assessment of change of direction speed, Y Balance Test™ (YBT™) for assessment of dynamic balance, force-velocity test on a stationary cycle ergometer test for peak and relative power per kilogram body mass, lateral box jump during 90 s for evaluation of performance capability during physiological demands comparable to alpine skiing, and a running treadmill test for direct assessment of V̇O_2peak_.

Maximal handgrip strength was quantified using a hydraulic hand dynamometer (Digital Hand Dynamometer Model SH5003, SAEHAN Corporation, Yangdeok-Dong, Masan, South Korea) according to the procedures described previously (7). The best performance (kg) out of three on each hand was registered.

The CMJ test was conducted using an optical timing system (MuscleLab 4020e (Ergotest Innovation, Stathelle, Norway). The best maximal jumping height (cm) out of three was registered.

The IAT was executed according to latest publication (27). The best performance time (s) out of three trials was registered using an infrared timing system (MuscleLab 4020e (Ergotest Innovation, Stathelle, Norway).

The 500 m rowing ergometer test was performed using a Concept2 Model D indoor rower (Concept2, Morrisville, Vermont, USA) according to the procedure described in (28). The time (s) to complete the test and the mean power (W) were recorded using the PM4 Performance Monitors built-in software.

The Force-velocity stationary cycle ergometer test was conducted using a Monark 894E Peak Bike (Monark Exercise, Vansbro, Sweden). Participants performed six maximal 10 s sprints from a standing start and against a breaking weight equivalent to 2, 4, 6, 8, 10% and 12% of their body weight. A restraint harness bolted to the bike frame was used to secure practitioners on the saddle. Sprints were conducted in a randomized order with ≥5 min of rest between each sprint. The best sprint on each load was recorded using Monark ATS Software (Monark Exercise, Vansbro, Sweden). The highest measured values [peak power output in absolute W (PPO) and peak power relative to body weight in W (RPP)] of all sprints were used in the statistical analysis.

The Y Balance Test was executed using a Y Balance Test Kit™ (Functional Movement, Danville, VA, USA) (29). Practitioners were allowed up to six trails in each direction on both feet before the commencement of the formal test. The best reach distance (cm) out of three attempts in each direction was registered.

The lateral box jump test was performed using a wood-frame box 40 cm high by 60 cm long by 50 cm wide (30). Participants were allowed a 20 s familiarization trial before the commencement of the formal test. From the top of the box and on the command “GO”, participants were instructed to jump down on one side, back up and down on the other side of the box as many times as possible for 90 s. The total number of landings (n) on top of the box with both feet simultaneously was registered.

The treadmill running test for V̇O_2peak_ was carried out according to the procedures described previously (7). The highest mean V̇O_2_ during 30 s recording was considered maximum and registered as V̇O_2peak_.

### 2.6 Statistical analysis

Data used in the statistical analysis were compiled of body composition measurements and physiological performance test results (X-variables) and FIS-ranking (Y-variables) during the years 2013–2017 seasons. As stated, PCA was used to identify representative body composition variables. Hotelling's T2 and DModX plots were used for the identification of outliers. Orthogonal Projections to Latent Structures (OPLS) were used to investigate regression (R^2^) and cross-validated prediction (Q^2^) of Y-variables (FIS-ranking in SL and GS) based on included X-variables. Cross-validation and permutation were conducted to validate the generated OPLS models.

For a simplified description of PCA and OPLS, we refer to previous work by our group (7).

Shared and Unique Structures (SUS)-plots were generated using individual OPLS models from four top-ranked skiers. The SUS-Plot is a scatter plot of the p(corr) (1) vector from two separate OPLS models (31). If two OPLS models have similar profiles (they capture similar relationships between the X-variables and the single Y-variable), the X-variables will line up along the diagonal, running from the lower left corner to the upper right corner. Such variables represent the shared structure among the two compared OPLS models. Conversely, X-variables not located along this thought diagonal, e.g., in the upper left corner, represent structures unique to either of the two models compared (extracted from the SIMCA handbook 2019-03-21) (32).

SIMCA's built-in test for normality was used to determine whether the included physiological testing results were significantly different from a normal distribution. Variables with significantly skewed distributions: stature (cm), right handgrip strength (kg), YBT™ right posteromedial (cm) and YBT™ left posterolateral (cm). Skewed variables were log10 transformed. Data was univariate scaled (UV) and analyzed in SIMCA 15.0 (Sartorius Stedim Data Analytics AB, Umeå, Sweden).

Data is presented as Mean ± SD if not otherwise indicated.

## 3 Results

**Figure 1:**
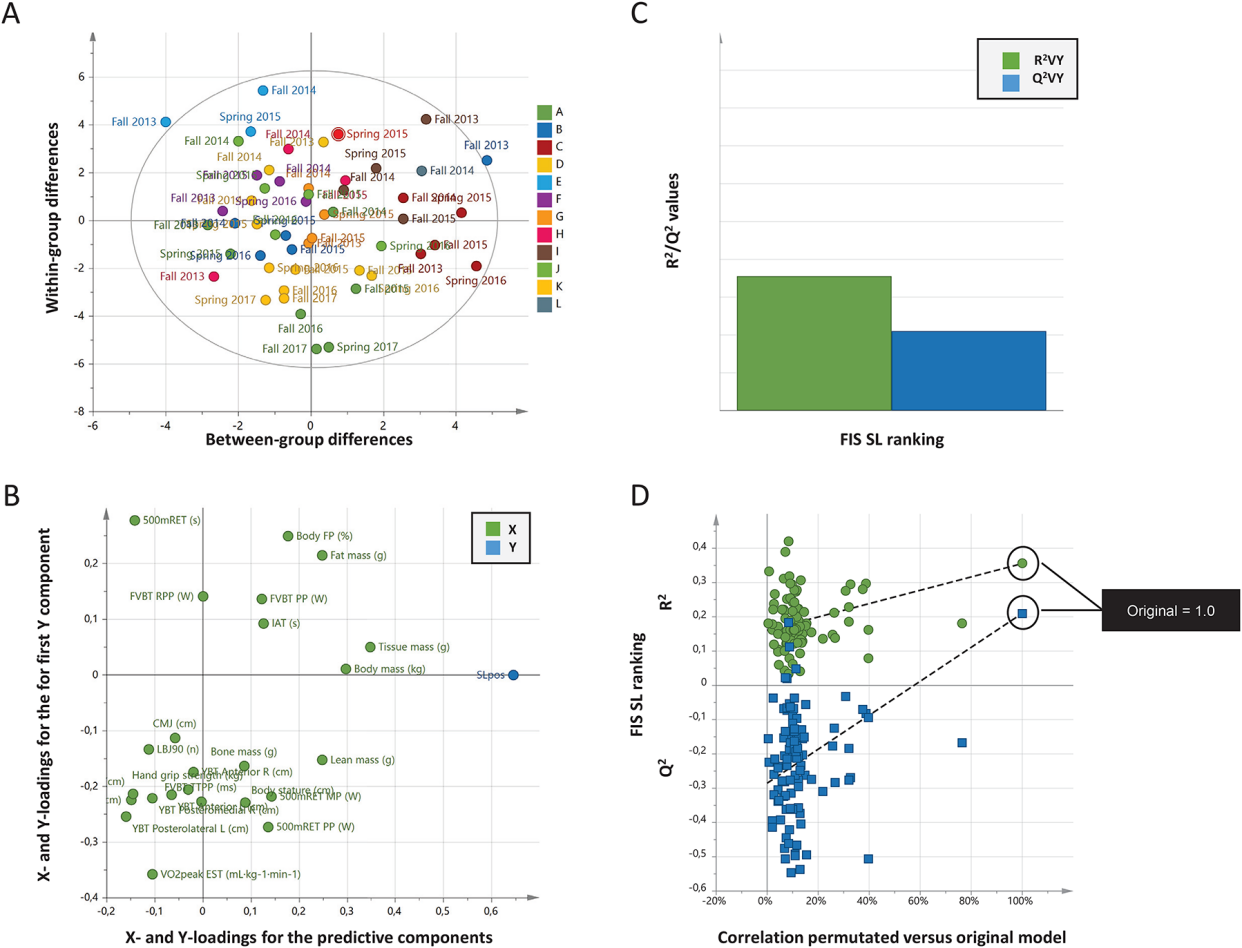
**(A–D)** Correlations between physiological testing results and FIS Slalom points (rankings). Data from 12 female world-class alpine skiers between the years 2013 and 2017. **(A)** Orthogonal projections to latent structures (OPLS) scatter plot of the scores t1 vs. t1o separates individual testing results over time. Letters A to L in the legend indicate each individual skier, and testing time is labelled in the plot. Ellipse: Hotelling's T^2^ (95%). **(B)** The loading scatter plot (X = 24, Y = 1; *n* = 12) visualizes correlations between variables. Physiological testing results and FIS points (rankings) located in the same part of the loading plot are correlated. The horizontal axis displays the X- and Y- loadings of the predictive component, and the vertical axis the X- and Y-loadings for the first Y-orthogonal component. A high value (max = 1) means that the component is aligned with the original variable, a value close to zero shows that it has no influence. A low value (min = –1) indicates an opposite influence. **(C)** X/Y overview plot shows the cumulated R^2^ and Q^2^ values for the model. **(D)** Cross-validation by repeated (100) permutations. The plot indicates the risk that the OPLS model is spurious, i.e., the model just fit the training set well but does not predict Y well for new observations. Goodness-of-fit (R^2^ and Q^2^) of the original model is compared with the goodness-of-fit of models based on data where the order of the Y observations has been randomly permuted, whereas the X matrix has been kept intact. For the selected Y-variable (Slalom), on the vertical axis for both models, the values of R^2^ and Q^2^ for the original model (far to the right) and of the Y-permuted models further to the left. The horizontal axis shows the correlation between the permuted Y-vectors and the original Y-vector for the selected Y. The original Y correlates 1.0 with itself, defining the high point on the horizontal axis. The plots above indicate that the original models are valid. The criteria for validity are that all blue Q^2^ values to the left are lower than the original points to the right, or the blue regression line of the Q^2^ points intersects the vertical axis (on the left) at or below zero. Consensus: When applying test batteries on a group level, no individual testing result, single or in combinations, could predict competitive performance measured as FIS ranking. The observed correlation between actual and predicted FIS ranking **(B)** display a valid model **(C,D)** but with low predictive power **(C)**.

**Figure 2:**
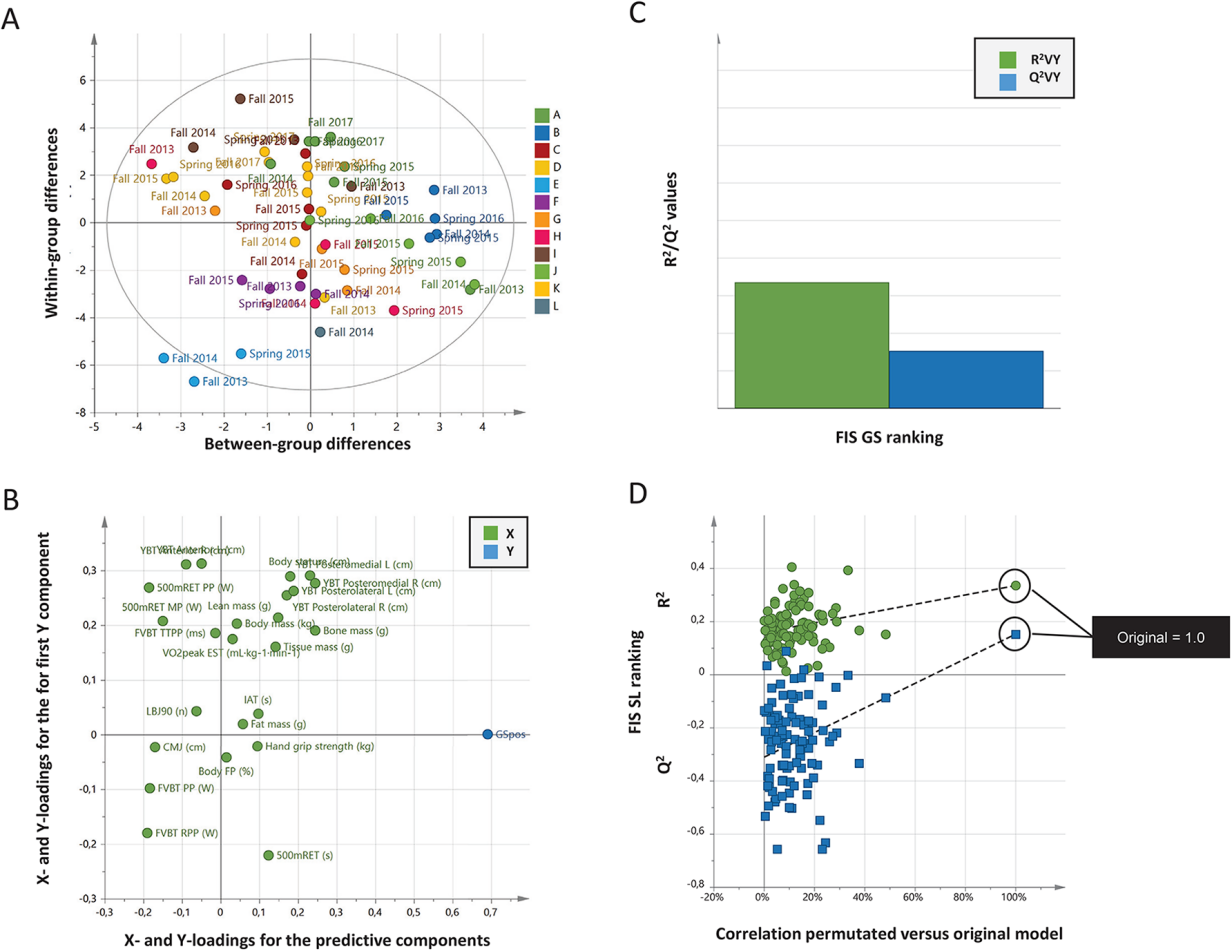
**(A–D)** Correlations between physiological testing results and FIS giant slalom points (rankings). Data from 12 female world-class alpine skiers between the years 2013 and 2017. **(A)** Orthogonal projections to latent structures (OPLS) scatter plot of the scores t1 vs. t1o separates individual testing results over time. Letters A to L in the legend indicate each individual skier, and testing time is labelled in the plot. Ellipse: Hotelling's T2 (95%). **(B)** The loading scatter plot (X = 24, Y = 1; *n* = 12) visualizes correlations between variables. Physiological testing results and FIS points (rankings) located in the same part of the loading plot are correlated. The horizontal axis displays the X- and Y- loadings of the predictive component, and the vertical axis the X- and Y-loadings for the first Y-orthogonal component. A high value (max = 1) means that the component is aligned with the original variable, a value close to zero shows that it has no influence. A low value (min = –1) indicates an opposite influence. **(C)** X/Y overview plot shows the cumulated R^2^ and Q^2^ values for the model. **(D)** Cross-validation by repeated (100) permutations. The plot indicates the risk that the OPLS model is spurious, i.e., the model just fit the training set well but does not predict Y well for new observations. Goodness-of-fit (R^2^ and Q^2^) of the original model is compared with the goodness-of-fit of models based on data where the order of the Y observations has been randomly permuted, whereas the X matrix has been kept intact. For the selected Y-variable (Giant Slalom), on the vertical axis for both models, the values of R^2^ and Q^2^ for the original model (far to the right) and of the Y-permuted models further to the left. The horizontal axis shows the correlation between the permuted Y-vectors and the original Y-vector for the selected Y. The original Y correlates 1.0 with itself, defining the high point on the horizontal axis. The plots above indicate that the original models are valid. The criteria for validity are that all blue Q^2^ values to the left are lower than the original points to the right, or the blue regression line of the Q^2^ points intersects the vertical axis (on the left) at or below zero. Consensus: When applying test batteries on a group level, no individual testing result, single or in combinations, could predict competitive performance measured as FIS ranking. The observed correlation between actual and predicted FIS ranking **(B)** display a valid model **(C,D)** but with low predictive power **(C)**.

No multivariable model (OPLS) could accurately predict (i.e., all have low Q^2^) competitive performance over time in SL and GS among elite female alpine skiers, based on included (Table 1) physiological testing results (Figures 1A–D, 2A–D).

Our stated research hypothesis is thus rejected.

Generated OPLS loading plots (Figures 1B, 2B) indicate separated clustering of FIS points (ranking) and physiological testing results (i.e., X and Y are not located in the same area of the plot). Goodness of regression (R^2^) and goodness of prediction (Q^2^) for FIS points (ranking) displayed in the X/Y overview plots (Figures 1C, 2C) indicate a low predictive power (R^2^ = 0.39–0.40, Q^2^ = 0.21–0.15) for SL and GS, respectively. Cross-validations by permutation (Figures 1D, 2D) confirm the validity of generated models. In this context, a non-significant model can be valid as being non-significant.

**Figure 3:**
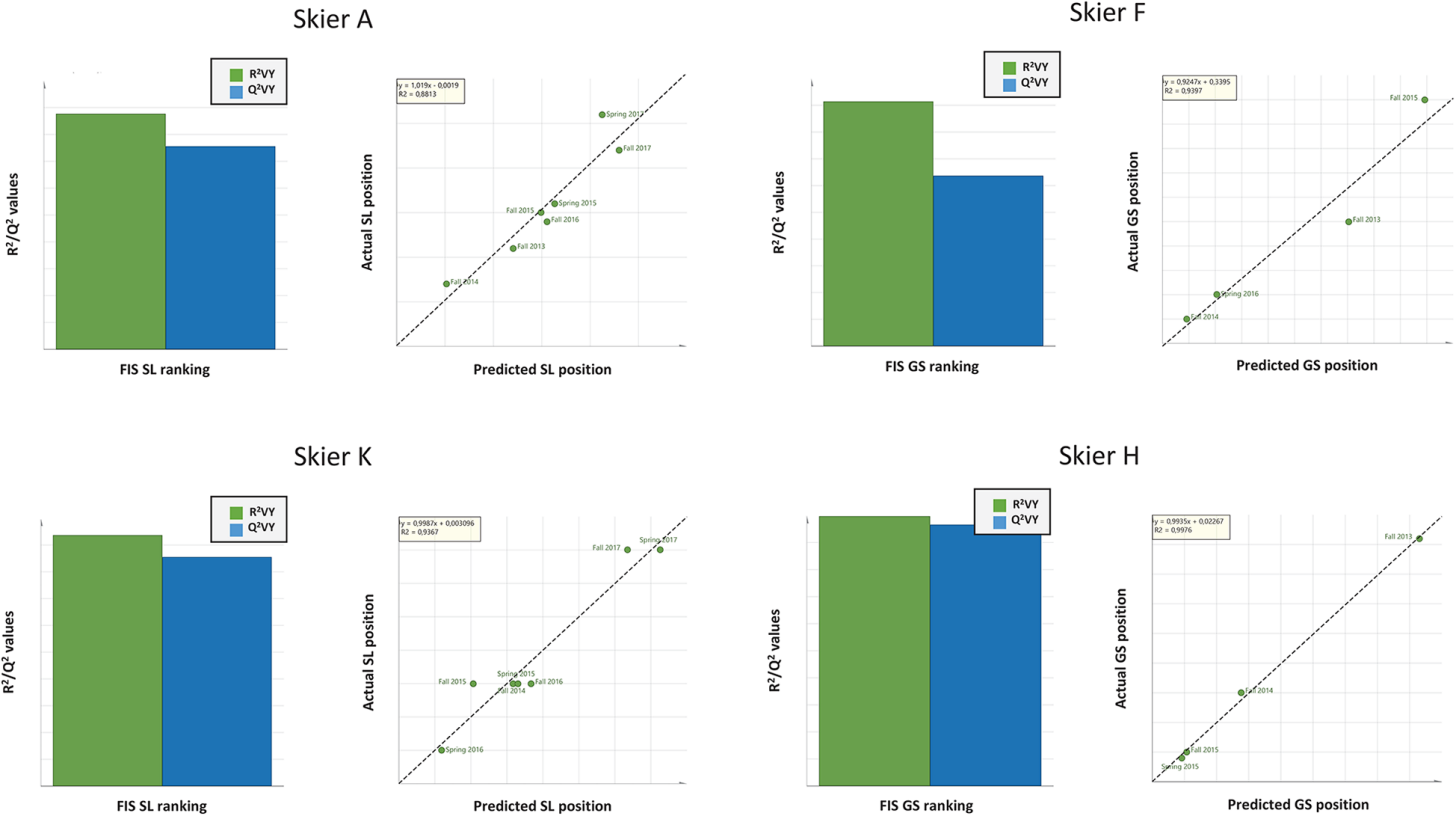
The X/Y overview plots (left hand side) show the cumulated R^2^ and Q^2^ values, exemplified from four individual athlete's models over time (slalom: A and K, giant slalom; H and F). These four skiers are all top ranked in the world, in their respective discipline. In models for Slalom, each athlete was tested seven times over the course of 4 years, with 23 different testing variables recorded each time (X = 23). In models for Giant Slalom, each athlete was tested four times over the course of 4 years, with the same 23 testing variables as in Slalom recorded. The regression plots (right hand side) display the observed vs. predicted values of the selected Y-variable. The RMSEE (Root Mean Square Error of Estimation) in the legends indicates the fit of the observations to the model (absolute measure). For ethical reasons and in order to protect the participants from identification, the x- and *y*-axis scaling were removed from each regression model. Consensus: When repeatedly recording the same physiological testing results, individual models (profiles) can be generated for high probability prediction of future competitive performance. This is contrast to when these same tests are applied on a group level, giving unreliable models.

**Figure 4:**
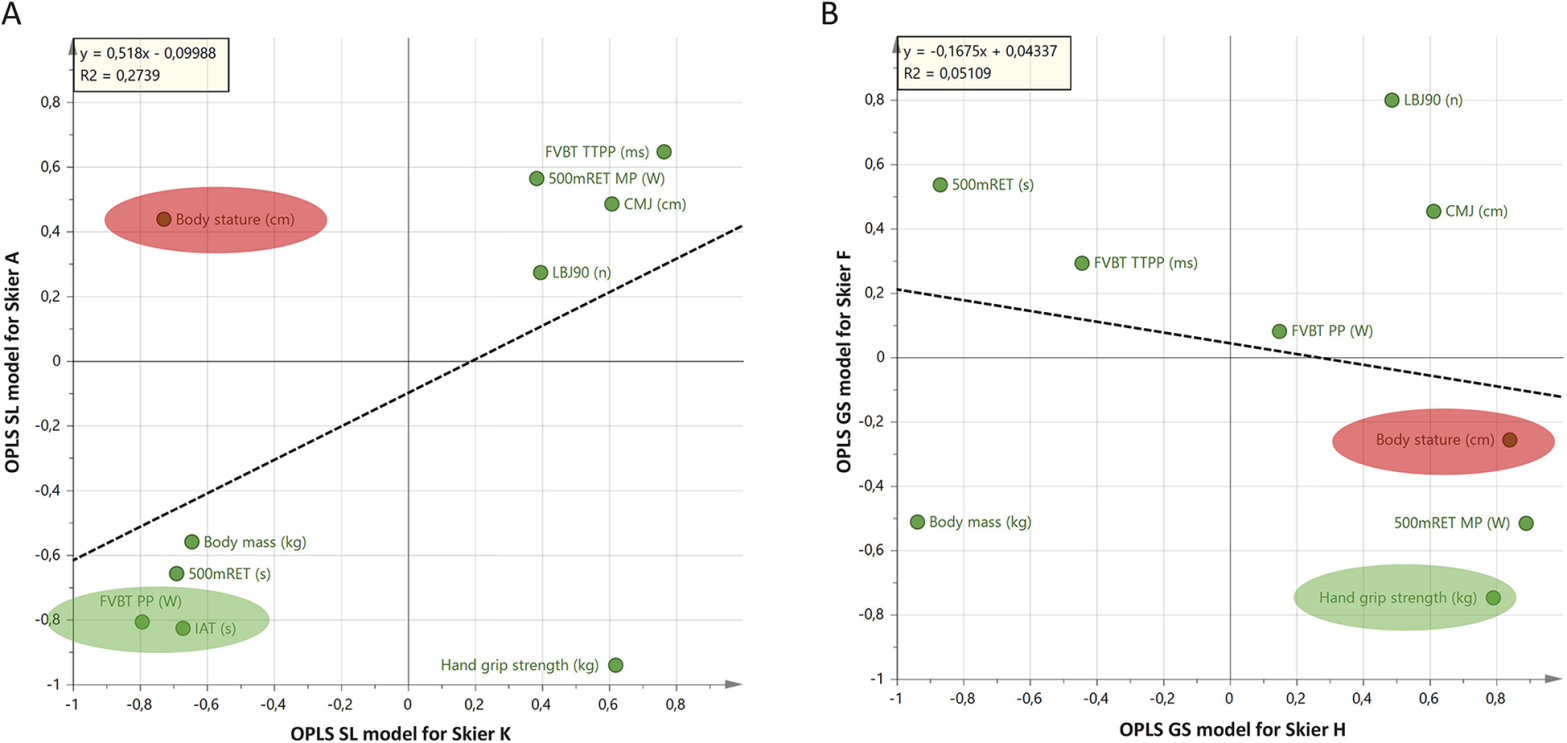
Shared and unique structures (SUS)-plot analyses from individual OPLS models exemplify individual differences in physical profiles of elite female alpine skiers. **(A)** Comparing individual profiles of skiers, A and K. The plot compares physiological testing results contributing to the model for Skier A [y-axis; 1 + 1 components; R2 = 0.88, Q2 = 0.76, p(CV-ANOVA) = 0.19] with that of the model for Skier K [x-axis; 1 + 1 components; R^2^ = 0.94, Q^2^ = 0.85, p(CV-ANOVA) = 0.87]. **(B)** Comparing individual profiles of skiers H and F. The plot compares the test results contributing to the model for Skier H [y-axis; 1 + 1 components; R^2^ = 0.99, Q^2^ = 0.96, p(CV-ANOVA) = 1.0] with that of the model for Skier F [x-axis; 1 + 1 components; R^2^ = 0.91, Q^2^ = 0.64, p(CV-ANOVA) = 1]. Both models indicate that each skier's competitive performance (FIS-rank) for Slalom and Giant Slalom depend on partially different physiological qualities. The physiological testing results located in the corners are important for both compared skiers, while variables located close to the X- and *Y*-axis are not important, or important for only one of the skiers. An inverse relationship exists if variables are located in the upper left and lower right corners. That is, high value is important for one individual, while a low value is important for the other. *Green ellipse* exemplifies one variable important for both individuals. *Red ellipse* exemplifies one variable more important for one individual compared to the other. Consensus: This analysis helps in understanding the unique and shared physiological factors that contribute to the performance of elite skiers, allowing for more personalized training and performance optimization. *Different Physiological Qualities:* Each skier's performance depends on different qualities. Variables located in the corners of the SUS-plot are crucial for both skiers being compared, while those near the axes are either not important or only important for one skier. *Inverse Relationships:* Variables in the upper left and lower right corners of the plot indicate an inverse relationship, meaning a high value is important for one skier, while a low value is important for the other. *Ellipses:* The green ellipse highlights variables important for both skiers, while the red ellipse shows variables more important for one skier compared to the other.

Data from competitive performance and physiological testing results recorded over time can be used to generate valid models for individual skiers (Figure 3) with higher predictive power (R^2^ = 0.88–0.99 and Q^2^ = 0.64–0.96) compared to when using the same data on a group level. Individual variation in physiological testing results, with similar/unchanged FIS points (ranking) (partially indicated by Figures 1A, 2A), is the main reason for the lack of high predictive models (R^2^/Q^2^ >0.6) on group level. To exemplify the unique individual profiles of equally top-ranked skiers, individual OPLS models for Slalom and Giant Slalom were generated and subsequently compared with the Shared and Unique Structures (SUS)-plot analysis (Figures 4A,B). Both SUS-plots indicate that each skier's competitive performance [FIS points (ranking)] for Slalom and Giant Slalom depends on individual, partially different physiological qualities.

## 4 Discussion

The outcomes of this investigation reveal that the incorporated tests yielded valid OPLS models for both slalom (SL) and giant slalom (GS) (Figures 1C,D, 2C,D), but models failed to withstand validation. Specifically, the models exhibited low predictive power for competitive performance at a group level (Figures 1C, 2C). In other words, models are true only for the dataset in which they are trained, but not applicable to a novel dataset. These findings are partially discordant with our previous research, in which no valid models for the investigated disciplines could be generated (6, 7), yet they converge in that all models demonstrated low predictive power (i.e., low Q^2^).

Consequently, these results imply that the competitive performance of elite alpine skiers cannot be predicted at a group level, regardless of the physiological performance tests employed.

In contrast, when physiological testing results and competitive performance data are analyzed at an individual level, robust models with high predictive power for competitive performance can be generated (Figures 3A–D). These findings corroborate the notion that multiple physiological qualities (e.g., muscle strength, balance, anaerobic and aerobic capacity) contribute to the competitive performance of alpine skiers (11), but also highlight that the relative importance of these qualities varies significantly between individuals. In the present study, FVBT PP (W) and IAT were found to be crucial for skiers A and K, whereas Body Stature was only significant for Skier A. Hand Grip Strength was important for skiers F and H, while Body Stature was only relevant for Skier H and A (Figure 4A). Historically, physiological performance research in alpine skiing has primarily focused on distinguishing between athletes at different performance levels (an indirect measure of performance differences) (16 33–35) or attempted to correlate physiological variables with ranking data (18, 30) and time trials (16, 17). Some studies have also used dubious statistical methods without validation of results (16, 30).

Our results indicate that normative data and non-validated, generalized research results are of limited use for the absolute top athletes.

Overall, our present results may provide important implications when implementing physiological performance tests for alpine skiers. While our previous studies showed that the included tests were unable to generate valid predictive models, our new results suggest that the problem may lie in how data are analyzed. Specifically, the new tests implemented in this study generated valid OPLS models for both SL and GS but also had low predictive power for competitive performance on a group level. Therefore, rather than implementing new tests to generate models with higher predictive power, it may be necessary to re-evaluate the analytical techniques used in this context.

Undoubtedly, elite performance in alpine skiing is influenced by multiple interdependent physiological qualities and other factors, including skiing technique, equipment, and mental dimensions (8, 10, 36).

A more extensive test battery, encompassing all these factors, may improve the test battery's predictive power regarding individual performance, even when applied at a group level.

Accordingly, future studies should adopt a more comprehensive and holistic approach to testing, with the aim of creating reliable and valid tests for the prediction and evaluation of competitive performance in alpine skiing.

Of outermost importance is to validate models with independent data, not used in the original model.

Also, the same test battery should be administered repeatedly over time rather than conducting new tests each year, as this approach may facilitate individual profiling and enable the identification of discriminatory variables for each athlete.

Moreover, other analytical methods, such as machine learning (ML) and artificial intelligence (AI), may mitigate the challenges associated with predicting group-level alpine skiing performance by analyzing large amounts of data from various sources, including different physiological and performance metrics, as well as environmental and race-specific factors (37). As a result, these methods can identify patterns and relationships difficult even for human experts to detect, possibly providing a more accurate and comprehensive assessment of an athlete's performance potential. In addition, ML and AI can continuously learn and improve from new data, leading to more accurate predictions and personalized training recommendations for individual athletes as they progress (37). As such, these analytical methods have the potential to enhance the accuracy and validity of the predictive models used in alpine skiing, enabling coaches and athletes to make more informed decisions regarding training and competition strategies over time.

To our knowledge, this is the first study using multivariable statistical methods to create individual profiles of world-class alpine skiers. However, while the presented results may provide valuable insights regarding its potential, further studies are needed to validate the approach using data sets from other sports. As such, the generalizability of this method can hopefully be determined and, therefore, also help to gain a more comprehensive understanding of its ability to predict performance in competitive sports.

In future studies, a more holistic approach for performance in alpine skiing should be considered, possibly including other variables than physical tests. Also, a defined sport performance parameter should always be included.

## 5 Practical implementations

The practical implications of these findings offer several important considerations for the development of performance testing and predictive modeling in competitive alpine skiing:

### 5.1 An individualized training and testing approaches

This investigation underscores the limited predictive power (prognostic capacity) of physiological tests assessed at the group level for predicting alpine skiing performance, whereas individual-level analysis yields highly predictive models for competitive performance. These findings imply that performance testing and training protocols should be customized to accommodate the unique characteristics of each athlete, emphasizing the superiority of personalized training regimens over standardized approaches.

Furthermore, coaches and trainers may derive benefits from examining individual physiological profiles to identify the specific variables that exert a profound impact on each athlete's performance, thereby informing the development of targeted training interventions.

### 5.2 Reevaluation of statistical and analytical methods

The findings of this study suggest that the primary obstacle in predicting group-level performance may reside in the analytical methodologies employed, rather than the tests themselves. This underscores the necessity for refining and advancing the methods utilized to analyze (sport)performance data. Traditional statistical models may require supplementation or replacement with more sophisticated approaches, such as ML and AI, which are capable of handling larger and more complex datasets, thereby potentially enhancing predictive accuracy. The intricacy of analyzing test data to predict performance is underscored by our investigation.

Given the absence of a significant effect of any test on predicting performance at the group level, an examination of the relative importance of specific tests across different seasons is rendered moot.

### 5.3 Comprehensive test batteries

Because alpine skiing performance is influenced by multiple interdependent factors — such as technique, equipment, and psychological aspects — future performance testing should integrate a broader range of variables.

Developing a holistic test battery including both physiological and non-physiological factors could enhance the predictive power for both individual and group-level performance.

### 5.4 Continuous monitoring and longitudinal testing

The study suggests that conducting repeated tests over time using the same battery, rather than introducing new tests annually, could help track individual progress and identify key performance variables for each skier. This approach would support continuous performance monitoring and allow for more accurate profiling of athletes.

### 5.5 Use of ML and AI

Incorporating ML and AI into performance analysis offers the potential to improve prediction models. These technologies can detect complex patterns in performance data and continuously adapt as new data are collected, allowing for more personalized and precise training recommendations. This could lead to more informed decision-making by coaches and athletes regarding training and competition strategies.

### 5.6 Validation of models and data use

The study emphasizes the importance of validating predictive models with independent data not used in the original model development. Validation ensures that the models are robust and applicable beyond the initial test group, increasing their reliability when applied in practical settings.

### 5.7 Potential application in other sports

While this study focuses on alpine skiing, the methods used could have broader applications in other sports. The approach of using individualized data analysis and advanced statistical techniques could improve performance predictions in various competitive sports, provided future research validates its generalizability.

In summary, these findings encourage a shift from generalized testing to more personalized and data-driven approaches in elite alpine skiing, emphasizing continuous individual profiling, advanced analytics, and a broader range of performance factors.

## Data Availability

The raw data supporting the conclusions of this article will be made available by the authors, without undue reservation.
